# Facing Contrast-Enhancing Gliomas: Perfusion MRI in Grade III and Grade IV Gliomas according to Tumor Area

**DOI:** 10.1155/2014/154350

**Published:** 2014-04-03

**Authors:** Anna Luisa Di Stefano, Niels Bergsland, Giulia Berzero, Lisa Farina, Elisa Rognone, Matteo Gastaldi, Domenico Aquino, Alessandro Frati, Francesco Tomasello, Mauro Ceroni, Enrico Marchioni, Stefano Bastianello

**Affiliations:** ^1^Neuro-Oncology Unit, C. Mondino National Neurological Institute, 27100 Pavia, Italy; ^2^Department of Brain and Behavioral Sciences, University of Pavia, 27100 Pavia, Italy; ^3^Magnetic Resonance Laboratory, IRCCS Don Gnocchi Foundation, 20148 Milan, Italy; ^4^Neuroscience Consortium, University of Pavia, Monza Policlinico and Pavia Mondino, 27100 Pavia, Italy; ^5^Neuroradiological Department, C. Mondino National Neurological Institute, 27100 Pavia, Italy; ^6^General Neurology Unit, C. Mondino National Neurological Institute, 27100 Pavia, Italy; ^7^Department of Neuroradiology, IRCCS Foundation Neurological Institute C. Besta, 20133 Milan, Italy; ^8^Department of Neurosurgery, IRCCS Neuromed, Pozzilli (IS), University of Rome La Sapienza, 00185 Rome, Italy; ^9^Department of Neurosurgery, University of Messina, 98122 Messina, Italy

## Abstract

Tumoral neoangiogenesis characterizes high grade gliomas. Relative Cerebral Blood Volume (rCBV), calculated with Dynamic Susceptibility Contrast (DSC) Perfusion-Weighted Imaging (PWI), allows for the estimation of vascular density over the tumor bed. The aim of the study was to characterize putative tumoral neoangiogenesis via the study of maximal rCBV with a Region of Interest (ROI) approach in three tumor areas—the contrast-enhancing area, the nonenhancing tumor, and the high perfusion area on CBV map—in patients affected by contrast-enhancing glioma (grades III and IV). Twenty-one patients were included: 15 were affected by grade IV and 6 by grade III glioma. Maximal rCBV values for each patient were averaged according to glioma grade. Although rCBV from contrast-enhancement and from nonenhancing tumor areas was higher in grade IV glioma than in grade III (5.58 and 2.68; 3.01 and 2.2, resp.), the differences were not significant. Instead, rCBV recorded in the high perfusion area on CBV map, independently of tumor compartment, was significantly higher in grade IV glioma than in grade III (7.51 versus 3.78, *P* = 0.036). In conclusion, neoangiogenesis encompasses different tumor compartments and CBV maps appear capable of best characterizing the degree of neovascularization. Facing contrast-enhancing brain tumors, areas of high perfusion on CBV maps should be considered as the reference areas to be targeted for glioma grading.

## 1. Introduction

Gliomas are the most common brain primary neoplasms and are classified based on histologic parameters including atypia, vascular endothelial proliferation, necrosis, and mitosis [1, 2]. A common histopathological characteristic for both grade III and grade IV glioma is vascular endothelial proliferation,which is known to correspond to blood brain barrier disruption and tumoral neoangiogenesis [3, 4] as evidenced by contrast-enhancement observed using conventional Magnetic Resonance Imaging (MRI).Although neuroradiological necrosis is a hallmark of glioblastoma (GBM; grade IV glioma), it is not a constant finding. Therefore it can be difficult to distinguish between grade III and grade IV gliomas using conventional MRI.

Dynamic Susceptibility Contrast (DSC) Perfusion-Weighted Imaging (PWI) measures concentration of a paramagnetic contrast material in the organ, providing reliable information on blood flow and vascularization. Among other perfusion parameters such as Cerebral Blood Flow (CBF) and Mean Transit Time (MTT), Cerebral Blood Volume (CBV) was shown to best correlate with tumoral neoangiogenesis and subsequently glioma grading. In particular, Relative Cerebral Blood Volume (rCBV), reflecting increased capillary density, was shown to be significantly higher in high grade gliomas (grade III and grade IV gliomas) than low grade gliomas (grade II glioma) and with higher values in grade IV [5].

Although rCBV increase is recognized as a surrogate marker of malignancy [6–8], differentiation between grade III and grade IV glioma is not consistently reproducible [8–11].

Furthermore, rCBV increases in glioblastoma multiforme (GBM) peritumoral area [10] and the peculiar pattern of rCBV increase surrounding glioblastoma contrast-enhancing tumor bed [12, 13] raises the question of where measuring rCBV values may best distinguish between grade III and grade IV gliomas.

In this study we investigated rCBV differences in patients with grade III and grade IV glioma using a Region of Interest- (ROI-) based method in three different tumor areas: the contrast-enhancing area, the nonenhancing tumor, and the high perfusion area on CBV map.

The aim of the study was to determine which tumor compartments showed rCBV differences related to glioma grade.

## 2. Materials and Methods

We examined perfusion MRI from 21 patients affected by histologically proven high grade gliomas: fifteen patients were affected by grade IV glioma and 6 patients by grade III glioma.

Twelve patients underwent a DSC perfusion examination before surgery and 9 with residual tumor were examined after surgery after a median of 6.8 months. All patients undergoing perfusion after surgery had residual tumor and were not in progression at the time of DSC perfusion examination. All patients in this study were affected by primary disease and in particular grade IV glioma patients were all affected by primary GBM.

Demographical characteristics of patients are detailed in Table 1. Glioma grading was assessed by an experienced neuropathologist according to WHO criteria 2007 [2]. The study was approved by the local Institutional Review Board and all patients provided informed consent.

All participants were scanned using a 1.5T Philips Intera Gyroscan (Philips Medical System, Best, The Netherlands) with a maximum slew rate of 150 Tm^−1^ s^−1^ and a maximum gradient amplitude of 30 mT/m. All scans were performed using an 8-channel SENSE (sensitivity encoding parallel imaging) head coil.

The scanning protocol included the following.

An axial 2D spin-echo (SE) T2-weighted Fluid Attenuated Inversion Recovery (FLAIR) image: echo time (TE)/repetition time (TR)/inversion time (TI) = 140/11000/2800 ms, flip angle (FA) = 90°, echo train length (ETL) = 50, acquisition matrix = 256 × 188, FOV = 250 mm^2^ (for an in-plane resolution of 0.9 mm × 1.3 mm), slice thickness = 5 mm, gap = 1 mm, number of excitations (NEX) =2, and number of slices = 24.

An axial 2D SE T1-weighted image: TE/TR = 15/649.5 ms, FA = 90°, ETL = 1, acquisition matrix = 260 × 209, FOV = 250 mm^2^ (in-plane resolution of 0.9 mm × 1.2 mm), slice thickness = 5 mm, gap = 1 mm, NEX = 2, and number of slices = 24.

An axial 3D perfusion weighted gradient echo (GRE) sequence (Principles of Echo Shifting with a Train of Observations, PRESTO) for Dynamic Susceptibility Contrast MRI: TE/TR = 8/16.72 ms (effective T2 = 23.71 ms), FA = 7°, ETL = 7, acquisition matrix = 64 × 64, FOV = 220 mm^2^ (in-plane resolution of 3.44 mm × 3.44 mm), slice thickness = 3 mm, NEX = 1, and number of slices = 30 with 40 temporal localizations. This sequence was acquired with a standard dose of 0.2 mmol/Kg body weight of gadopentetate dimeglumine (Gd-DTPA) contrast agent (Gadovist) which was injected at a rate of 4 mL/s, followed by a 20 mL continuous saline flush. Using a 0.05 mmol/Kg dose, presaturation of the baseline signal prior to the PWI acquisition was done to reduce T1-effects as well as potential contrast leakage effects due to blood brain barrier disruption.

An axial 3D T1-weighted fast field echo (FFE) sequence after the PWI: TE/TR = 4.6/25 ms, FA = 30°, ETL = 1, acquisition matrix = 256 × 256, FOV = 250 mm^2^ (in-plane resolution of 0.98 mm × 0.98 mm), slice thickness = 1.6 mm, gap = 0, NEX = 1, and number of slices = 170.

Postprocessing was performed using Olea Medical PerfScape software (version 2.0). The DSC acquisition was corrected for patient motion using the built-in feature of PerfScape. The T1 SE, FLAIR, and T1 3D FFE images were then coregistered and resampled into the space of the DSC MRI. It is well known that disruption of the blood brain barrier, as is common in high grade tumors, can lead to inaccurate measures of CBV [14]. As such, the correction for leakage effects option in PerfScape was employed.

Relative CBV (rCBV) was calculated from three separate ROIs that were placed in three different compartments: the area of contrast-enhancement, the nonenhancing tumor, and high perfusion area seen on the CBV color overlay maps. In the contrast-enhancement area, necrosis was excluded from CBV measure. The nonenhancing surrounding tumor ROIs corresponded to areas of T2/FLAIR hyperintensity outside contrast-enhancement. We ensured that T2/FLAIR ROIs were not placed in areas of contrast-enhancement as all images were coregistered. Placement of ROIs on the CBV map was performed in high perfusion areas independently of the contrast-enhancement and T2/FLAIR ROI locations.

Morphology and size of the ROIs were constant (elliptical-40 mm² area) and the maximum value was recorded for each compartment according to Law et al. [15].

All values were normalized to a corresponding ROI placed in contralateral normal brain parenchyma. All ROIs were placed by two operators (A.D, N.B.) via consensus. Maximal rCBV values for each patient were averaged according to glioma grade.

Differences between grades and tumor areas were tested using* t*-test. ANOVA was used in order to compare rCBV values in different subgroups of patients. Contingency analysis was performed by Fisher's exact test. In all analyses we considered a *P* value of 0.05 (two-sided) as being statistically significant.

## 3. Results

Twelve patients underwent a DSC perfusion examination before surgery (3 patients affected by grade III glioma and 9 patients affected by grade IV glioma) and 9 patients with residual tumor were examined after surgery after a median of 6.8 months (3 patients affected by grade III glioma and 6 by grade IV glioma). Distribution of patients according to the timing of perfusion MRI (presurgery versus postsurgery) was not significantly different between grade III glioma and grade IV (*P* = 0.67).

In the grade III glioma subgroup, 2 patients were affected by anaplastic oligodendroglioma and 4 patients by anaplastic astrocytoma; in the grade IV glioma subgroup all patients were affected by glioblastoma.

Mean rCBV values from patients are detailed in Table 2. In the grade III glioma subgroup, mean rCBV was higher in the contrast-enhanced area than nonenhancing tumor (3.01 and 2.20, resp., *P* = 0.11); rCBV recorded in CBV map, independently of tumor compartment as seen on conventional MRI, was 3.78.

In glioma grade IV, mean rCBV was higher in contrast-enhanced area than nonenhancing tumor (5.58 and 2.68, resp., *P* = 0.04); the mean rCBV recorded in the CBV map was 7.51.

**Figure 1:**
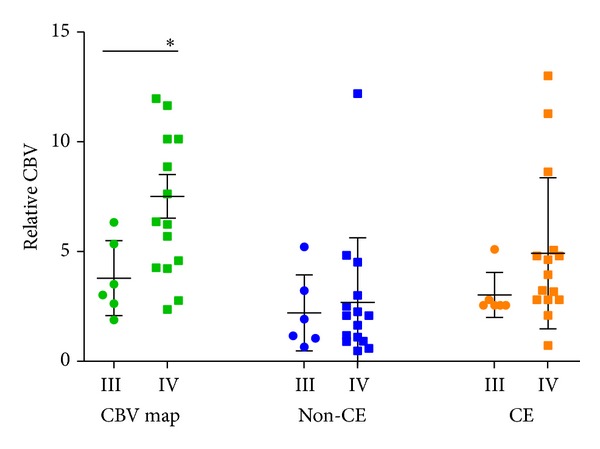
Scatter-plot diagram representing rCBV values according to grade and tumor area. Circles represent grade III, squares grade IV. Green color represents rCBV values measured in CBV map; blue color represents rCBV values measured in the nonenhancing tumor (Non-CE); orange color represents rCBV values measured in enhancing area (CE). Lines correspond to mean value and error bars to standard error of the mean. Only rCBV values measured in the high perfusion area in CBV map showed significant difference between grade III and grade IV gliomas.

Between glioma grade III and glioma grade IV, no significant differences in rCBV were observed in the contrast-enhancement area and in the nonenhancing tumor (*P* = 0.27 and 0.71, resp.). Inversely, mean rCBV was significantly higher in grade IV gliomas than in grade III (*P* = 0.036) in the high perfusion area of CBV map independently of tumor compartment, as seen on conventional MRI (Figure 1).

## 4. Discussion

Neovascular proliferation is a hallmark of malignant gliomas and PWI is useful in glioma grading through detection of vascular density and of the grade of tumor-associated neovascularization [7, 16].

The measure of rCBV is commonly used in order to predict glioma grade or to differentiate radionecrosis from tumor recurrence in a diagnostic setting. Several reports on rCBV increases in peritumoral area of glioblastoma [10, 13] have suggested that there is a mismatch between the extension of effective vascular proliferation and area of contrast-enhancement.

In this work we mapped rCBV maximal increase in two different compartments of glioma grade III and glioma grade IV—the contrast-enhancing area and nonenhancing tumor with a ROI-based method. rCBV was also recorded in high perfusion area of CBV map independently of corresponding tumor area on conventional MRI.

Values of rCBV recorded in this work are consistent with the other reports in the literature [6]. As expected we found a significantly higher rCBV in contrast-enhanced area than in nonenhancing tumor in the grade IV glioma subgroup.

Concerning rCBV differences according to the tumor grade, we did not find significant differences of rCBV values recorded in contrast-enhancing area or nonenhancing tumor between grades III and IV.

Only measures of rCBV in the high perfusion area on the CBV map showed a significant difference between grade III glioma and grade IV, with higher values in grade IV.

Taken together, these results support the idea that neoangiogenesis heterogeneously encompasses both contrast-enhancing and nonenhancing tumor areas. The contrast-enhancing areas appear to reflect a higher degree of neoangiogenesis, although the difference with respect to nonenhancing areas was significant only in grade IV glioma subgroup.

Interestingly, we did not find significant differences in maximal rCBV in neither of these two areas when comparing grades III and IV. This suggests that basing rCBV measurements on signal characteristics of conventional MRI may not be sufficient to distinguish between grade III and grade IV gliomas.

Glioblastoma has been shown to present with a more heterogeneous neovascularization than grade III glioma [12]. In particular glioblastoma present, more so than with lower grades, areas with low perfusion due to necrosis, area of focal rCBV increase, and also increased rCBV values in peritumoral normal-appearing parenchyma [14, 17].

**Figure 2:**
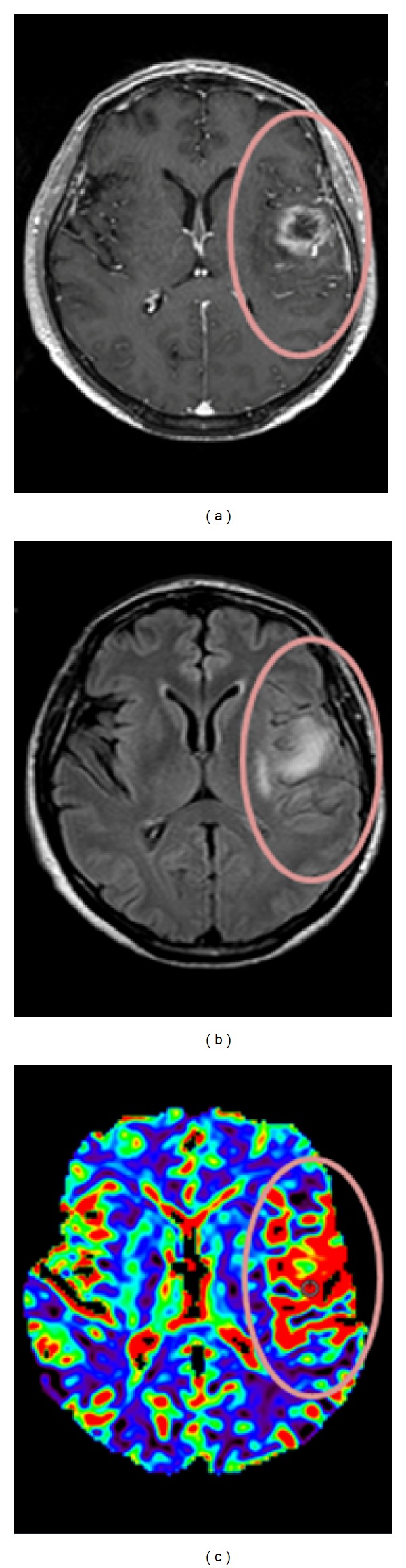
Axial coregistered contrast-enhanced axial T1-weighted image (a), FLAIR image (b), and CBV map (c) from a patient affected by glioma grade IV. In the CBV map (c) warmer colors indicate higher CBV values suggesting higher perfusion and neovascularization. Comparison of CBV map (c) and contrast-enhanced axial T1-weighted image highlights a mismatch area (surrounded by the circle) corresponding to the extension of the high perfusion area outside the contrast-enhancement: this indicates a more extensive neovascularization than that shown by conventional MRI (a, b).

In particular, a special pattern of rCBV increase in peritumoral area can occur in a “stripe like” fashion which has been termed a “striate sign.” This feature has been described as mostly represented in glioblastoma rather than lower grade gliomas and in particular with respect to grade III glioma [12, 13]. The same authors showed that this specific pattern of rCBV in peritumoral area was significantly associated with normalized choline increase and with the subsequent appearance of contrast-enhancement in the same area [12, 13]. A similar example of mismatch between high perfusion area from CBV map and contrast-enhancement observed in our patients is shown in Figure 2. Histopathologically, these patterns of rCBV may reflect diffuse migration of glioma cells along vascular channels of the white matter tracts spreading beyond the visible tumor border [18].

Taken together, these results support the hypothesis that only the rCBV map represents extensively the neovascular phenomena, its extension into apparently normal surrounding parenchyma, and its quantitative difference among glioma grades.

Limits of the study are the small sample size and potential sampling differences from the ROI-dependent method of measure which may increase interobserver variability. Nevertheless the latter is the most used in clinical routine. Additionally, all ROIs were placed in consensus by two authors. The fact that some patients were scanned before surgery and other patients afterwards presents an additional confound. However, we did not find any significant differences between the pre-/posttreatment groups (results not shown). Nevertheless, we cannot rule out the possibility that surgical intervention in some patients may have influenced the results.

In conclusion, maximal rCBV values measured directly on the CBV map seem to best characterize the extensive neoangiogenesis phenomena of high grade gliomas and quantitative difference of microvascular density between grade III and grade IV glioma. Such measurements should be considered as the reference map for glioma grading and potentially for serial measures of rCBV modification during antiangiogenic treatment.

## Figures and Tables

**Table 1:** Patients' clinical data and tumor diagnosis.

WHO glioma grade	Number of patients	Age (years) (median, range)	Sex ratio (male/female)
III	6	49 (24–66)	6.0
IV	15	63 (23–80)	1.5

**Table 2:** Mean rCBV values according to histological grading. Relative Cerebral Blood Volume (rCBV) was measured with the ROI-based approach in three distinct areas: the high perfusion area on CBV map (“CBV map” in the table), the contrast-enhanced area (“CE” in the table), and the nonenhancing tumor (“Non-CE” in the table). Only rCBV values measured in the high perfusion area in CBV map showed significant difference between grade III and grade IV gliomas.

WHO glioma grade	rCBV; mean (SD)					
	CBV map	*P*	CE	P	Non-CE	P
Glioma grade III	3.78 (1.70)	**0.036**	3.01 (1.02)	0.27	2.20 (1.73)	0.71
Glioma grade IV	7.51 (3.84)		5.58 (5.48)		2.68 (2.93)	
